# Supplementary material for: Does a Healthy Lifestyle Lower the Elevated Risk of Obesity Caused by Caesarian Section Delivery in Children and Adolescents?
Source: Nutrients. 2022 Aug 26;14(17):3528. doi: 10.3390/nu14173528 (PMC9460904; doi:10.3390/nu14173528)
Supplement: Supplementary file 1 [file nutrients-14-03528-s001.zip › nutrients-1857374-supplementary.pdf]

# Supplementary Materials

**Table S1** Output of logistic regression model with delivery mode and lifestyle as dichotomous determinants and product of delivery and lifestyle entered into the model. Outcome is obesity.

| Parameters | Estimate | SE | 95%CI of OR | | |
|---|---|---|---|---|---|
| | | | *OR* | Lower | Upper |
| Delivery | 0.587 | 0.033 | 1.80 | 1.69 | 1.92 |
| Lifestyle | 0.250 | 0.054 | 1.28 | 1.16 | 1.43 |
| Delivery × Lifestyle | -0.020 | 0.065 | 0.80 | 0.84 | 1.14 |
| Constant | -2.377 | 0.024 | | | |
| RERI(95%CI) | 0.18 (-0.12, 0.49) | | | | |

Abbreviations: SE, Standard Error; OR, Odds Ratio; RERI, Relative Excess Risk due to Interaction.
